# The role of sarcopenia in the development of early complications in patients with advanced epithelial ovarian carcinoma undergoing cytoreductive surgery: Sarcopenia in epithelial ovarian carcinoma undergoing cytoreductive surgery

**DOI:** 10.1007/s00423-025-03761-1

**Published:** 2025-06-13

**Authors:** Gökhan Coşkun, Ufuk Karabacak, Halil Türkan, Murat Can Mollaoğlu, Meriç Emre Bostancı, Kürşat Karadayı

**Affiliations:** 1 Department of General Surgery and Surgical Oncology, Tokat Gaziosmanpaşa University Faculty of Medicine, Tokat, Turkey; 2 Department of General Surgery, and Surgical Oncology, Ondokuz Mayıs University Faculty of Medicine, Samsun, Turkey; 3 Clinic of Surgical Oncology, Mehmet Akif İnan Education and Research Hospital, Şanlıurfa, Turkey; 4 Clinic of General Surgery and Surgical Oncology, İstinye University Gaziosmanpaşa Medikalpark Hospital, İstanbul, Turkey; 5 Clinic of Surgical Oncology, Ministry of Health Sivas Numune State Hospital, Sivas, Turkey; 6 Clinic of Surgical Oncology, İstinye University Gaziosmanpaşa Medikalpark Hospital, İstanbul, Turkey

**Keywords:** Sarcopenia, Ovarian cancer, Primary cytoreductive surgery, Postoperative complications

## Abstract

**Purpose:**

Sarcopenia has been identified as a prognostic factor in various cancers. The aim of the study is to investigate the role of sarcopenia in the development of early complications following Primary Cytoreductive Surgery in advanced stage ovarian cancer patients.

**Methods:**

A total of 57 patients who underwent Cytoreductive Surgery due to advanced stage ovarian cancer at the Surgical Oncology Clinic of…………. University Hospital between January 2013 and June 2021 were retrospectively analyzed. Routine preoperative Computed Tomography(CT) images were used to measure the cross-sectional areas of the paraspinal muscles, abdominal wall muscles at the L3 level in cm2. The Skeletal Muscle Index (SMI) and Psoas Muscle Index (PMI) values were calculated. Postoperative early complications were classified as Clavien-Dindo grade 3 and above major complications.

**Results:**

Based on SMI measurements, 23 patients with SMI < 38.5 were classified as sarcopenic, while 34 patients with SMI ≥ 38.5 were classified as nonsarcopenic; based on PMI measurements, 19 patients with PMI < 4.3 were classified as sarcopenic, while 38 patients with PMI ≥ 4.3 were classified as nonsarcopenic. Major complications occurring in the early postoperative period were significantly more common in the sarcopenic group. High Peritoneal Carcinomatosis Index (PCI) score and FIGO 3 C stage were associated with a significantly higher incidence of early complications. High PCI score and SMI < 38.5 value were identified as risk factors for postoperative early complications.

**Conclusions:**

The quantitative measurements of sarcopenia using routine CT imaging for preoperative staging are a useful and cost-effective method. Determining the sarcopenic status of patients before treatment offers an advantage in predicting treatment complications.

## Ethical approval

This study was approved by the Ethics Committee of Sivas Cumhuriyet University. (Approval number:2021-05/38, date:26.05.2021).

## Introduction

Epithelial origin gynecologic cancers are among the most common types of cancer in women. Globally, they rank fifth among the causes of death in women. Although survival rates have increased over the past decade, ovarian cancer still holds the highest mortality rate among gynecologic malignancies [1, 2]. The majority of ovarian cancer patients present with peritoneal metastasis at the time of diagnosis [3]. Treatment of ovarian cancer is based on the Fédération Internationale de Gynécologie et d’Obstétrique(FIGO) classification. Surgical intervention remains the gold standard in the management of advanced-stage Epithelial Ovarian Cancers with peritoneal spread. Primary Cytoreductive Surgery (PCS), aimed at achieving either no residual tumor or minimal residual tumor, is the primary treatment method contributing to overall survival in these cancers [4–6].

IntervalCytoreductive Surgery (ICS) following Neoadjuvant Chemotherapy is preferred when achieving R0 or R1 status seems unlikely. Some studies have shown no significant difference in terms of survival and early complications between PCS and ICS [7]. Various factors contribute to the development of early postoperative complications, including tumor-related factors and patients’ characteristic features [8].

Sarcopenia, one of the characteristic features of patients, refers to the progressive loss of muscle mass, muscle strength, and function over time. First described by Rosenberg in 1989, its clinical definition was established by Baumgartner et al. [9]. Sarcopenia is a multifactorial condition whose prevalence increases with age [10]. It is also associated with conditions such as heart failure, chronic kidney disease, malnutrition, and various cancers [11, 12]. Sarcopenia has been identified as a prognostic factor in several cancers, including pancreatic, hepatic, biliary tract, gastrointestinal, and urothelial cancers [13–16]. In a study on upper urothelial cancers, when the SMI cut-off was calculated as < 41 cm^2^/m^2^ in women and < 43 cm^2^/m^2^ in men, 58% of the patients were reported to have sarcopenia. In upper urothelial cancer, Sarcopenia has been reported to be an independent prognostic factor [14]. In a meta-analysis including 13 studies, the prevalence of sarcopenia was reported as 17–79%. In 7 studies of operated GIT and Hepatobiliary cancers, sarcopenia was reported to be associated with poor overall survival, independent of the tumor. It has been reported that it is associated with an increase in postoperative complications in colorectal cancer [15]. In another study, sarcopenia was detected in 21.3% of patients with advanced pancreatic cancer at the time of diagnosis. The SMI cut-off was calculated as < 42.2 cm^2^/m^2^ in male patients and < 33.9 cm^2^/m^2^ in female patients. During chemotherapy treatment, a 60.9% decrease in SMI was observed in males and 40.6% in females. This condition was reported to be a poor prognostic factor for overall survival [16].

However, relatively fewer studies have focused on sarcopenia in gynecologic cancers.

**Fig. 1:**
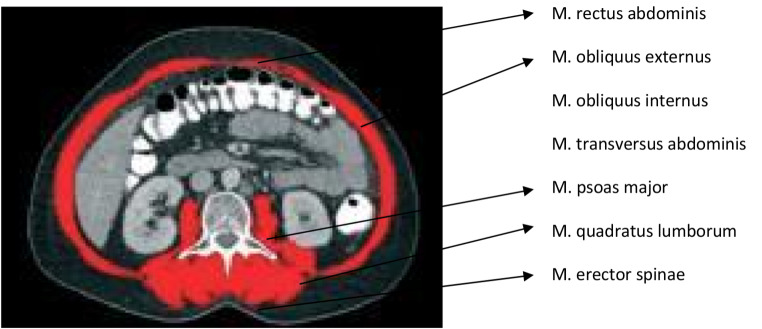
Total skeletal muscle area, Red painted (Abdominal CT, Axial Section, L3 level)

**Fig. 2:**
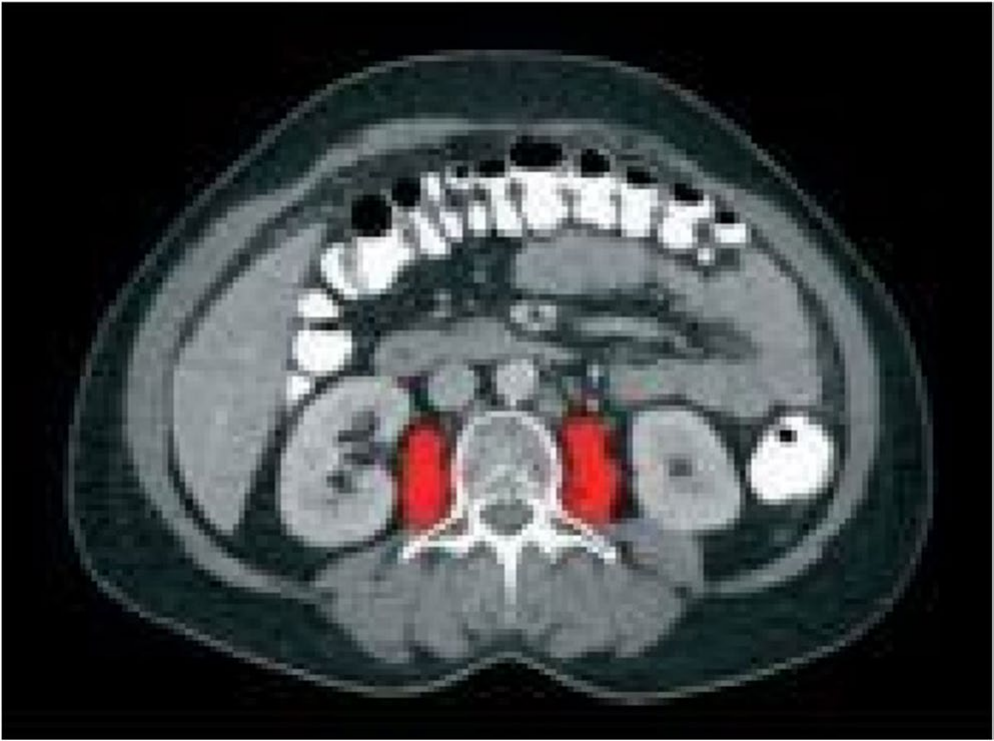
Psoas muscle area, Red painted (Abdominal CT, Axial Section, L3 level)

Cancer-associated sarcopenia is part of the cancer cachexia syndrome, whose diagnosis is based on either weight loss alone or a combination of muscle and weight loss. In advanced-stage ovarian cancers, weight loss may be masked due to the formation of intraperitoneal ascites. Therefore, quantitative measurement of skeletal muscle mass provides more accurate information regarding cancer cachexia. Magnetic Resonance Imaging and Computed Tomography (CT)imaging can be utilized for quantitative measurement of skeletal muscle mass [11–17]. In our retrospective study, CT-Imaging is routinely performed during staging and surgical planing, making it a readily available and reliable method (Figs. 1 and 2).

In this study, we investigated the role of sarcopenia in the development of early postoperative complications following Primary Cytoreductive Surgery in advanced-stage ovarian cancer patients.

Aim of the study should highlight the potential for sacropenia assessment to influence the preoperative optimization strategies.

## Materials and methods

This study was approved by the Ethics Committee of Sivas Cumhuriyet University. (Approval number:2021-05/38, date:26.05.2021).

### Study population

A retrospective analysis was conducted on 72 patients who underwent Primary Cytoreductive Surgery for advanced-stage serous ovarian cancer at the Surgical Oncology Clinic of……………University Hospital between January 2013 and June 2021. Abdominal CT images taken within 50 days prior to surgery were screened. Eight patients were excluded from the study due to either unavailability or inadequate quality of CT images. Five patients who received neoadjuvant chemotherapy were also excluded. Additionally, two patients with a second primary (metachronous) malignancy were excluded. CT images of the abdomen of 57 included patients were reviewed.

### Calculation of Skeletal Muscle Area, Skeletal Muscle Index, Psoas major muscles, and Psoas Muscle Index

In Axial sections of Abdominal CT at the L3 level were used to measure the cross-sectional areas (in cm^2^) of the right and left paraspinal muscles (Quadratus lumborum, Erector spinae, Psoas major), abdominal wall muscles (External oblique, Internal oblique, Transversus abdominis), and Rectus abdominis muscles. Measurements were performed using OsiriXsoftware 5.0. The areas of tissues with muscle density (between + 25 + 50 Hounsfield Unit) were calculated. Anatomically, it was confirmed that these areas were muscle groups with anatomical names. Measurements were verified by 2 authors.

The Skeletal Muscle Index (SMI) was calculated by normalizing the Skeletal Muscle Area (SMA) value to the square of the patient’s height. Additionally, the total cross-sectional area of the right and left Psoas major muscles (Psoas Muscle Area, PMA) was measured separately, and the Psoas Muscle Index (PMI) was calculated by normalizing it to the square of the patient’s height.

In the control group, consisting of 26 women without malignancy who underwent abdominal CT for reasons other than major abdominal surgery during the same period and in a similar age group, SMA, SMI, PMA, and PMI values were measured, and cut-off values were determined for SMI and PMI. Cut-off values of 38.5 cm2/m2 for SMI and 4.3 cm2/m2 for PMI were established. These measurements, although subject to variations based on race, environmental factors, and societal differences, are consistent with international consensus values (OECD 2020).

Among the patient group, 23 patients with SMI < 38.5 were categorized as sarcopenic, while 34 patients with SMI > = 38.5 were classified as nonsarcopenic. According to PMI measurements, 19 patients with PMI < 4.3 were considered sarcopenic, while 38 patients with PMI > = 4.3 were categorized as nonsarcopenic.

### The clinicopathological characteristics of patients

The clinicopathological characteristics of patients, including age, Body Mass Index (BMI), preoperative albumin, Hb, WBC, Plt, ASA score, FIGO stage, tumor grade, presence of ascites, operative time, intraoperative hemorrhage, Residual tumor status, length of hospital stay, Peritoneal Carcinomatosis Index (PCI) score, and intraperitoneal chemotherapy status, were examined.

Early postoperative complications (within the first 30 days) were classified according to the Clavien-Dindo scale. Complications graded as Clavien-Dindo 3 and above were considered major complications.

### Statistical analysis

Statistical analysis of the study was performed using SPSS software (Version 20, SPSS Inc., Chicago, IL, USA). Mann-Whitney U test and Logrank (Mantel-Cox) testiwas used to analyze variables. Survival curves wereprepared according to the Kaplan–Meier method and statistical differences were persuadedusing the log-rank test. Univariate and multivariate Cox-proportional hazard models wereused to evaluate risk factors. P value < 0.05was considered to indicate statistical significance.

## Results

### The statystical analyses of the clinicopathological characteristics of the patients

The demographic, clinical and histopathologic characteristics, laboratory results, surgery time, intraoperative hemorrhagiae, intraperitoneal chemotherapy, hospitalization time of all patients are shown in Table 1.

**Table 1:** The clinicopathological characteristics of the patients

Characteristics of patients	*n* (%)	Sarcopenic(SMI < 38,5)	Nonsarcopenic (SMI > = 38,5)	*P* value
Age average	55,4 (32–79)	55,8	55,2	0,846
BMI (kg/m2)	27,2 (20,2–34,3)	25,9	28,1	0,092
Obesity(BMI > 30)	16 (%28,0)	5 (%21,7)	11 (%32,3)	
ResidualtumorafterPCS				
0–2,5 mm	46 (%80,7)	18 (%78,2)	28 (%82,3)	0,229
2,5 mm–2 cm	8 (%14,0)	3 (%13,0)	5 (%14,7)	
> 2 cm	3 (%5,3)	1 (%4,3)	2 (%5,8)	
Histologicalgrade				
1	1 (%1,7)			
2	4 (%7,0)			
3	52 (%91,3)			
PeritonealCarcinomatosisindex	24,1 (14–30)	24,3	23,9	0,938
Ascit				
Positive	50 (%87,7)	20 (%87,0)	30 (%88,2)	0,421
Negative	7 (%12,3)	3 (%13,0)	4 (%11,8)	0,138
FIGO				
3A	25 (%43,9)	10 (%43,5)	15 (%44,1)	0,731
3B	5 (%8,7)	2 (%8,7)	3 (%8,8)	
3C	27 (%47,4)	11 (%47,8)	16 (%47,1)	
Albumingr/dl	37,2 (24,1–51,2)	35,3 (24,1–46,2)	38,4 (27,2–51,2)	0,398
WBC	7,8 (3,1–15)	7,9 (3,1–13,8)	7,7 (3,8–15)	0,483
PLT	217(122–526)	230 (132–526)	210 (122–486)	0,232
ASA				
1	6 (%11,5)			
2	12 (%21,0)			
3	39 (%68,5)			
Surgery time (min)	226,5 (110–300)	231 (130–280)	214,7 (110–300)	0,156
Intraoperativhemorajıa(ml)	440 (200–900)	497 (290–900)	425 (200–750)	0,094
Intraperı̇tonealChemotherapy (HIPEC+/- EPIC)	57 (%100)			
Length of hospitalization (days)	16,3 (5–88)	16,9 (5–49)	16,0 (7–88)	0,186

*BMI* Body mass index, *PCS* Primary Cytoreductive Surgery

### Comparision of Skeletal Muscle Area, Skeletal Muscle Index, Psoas major muscles, and Psoas Muscle Index with hospital length, operation time, intraoperative hemorrhage, and survival time

No statistically significant difference was observed in the median values of hospital length, operation time, intraoperative hemorrhage, and survival time among sarcopenia and nonsarcopenia patients across different SMI cutoff values (*p* > 0.05) (Table 2).

**Table 2:** Comparison of hospitalization time, operation time, intraop hemorrhage and survival time according to SMI cut-off value

	SMI Cutoff				*P* value*
	≥ 38,5		< 38,5		
	Ort. ± s. Sapma	Ort. (min. - maks.)	Ort. ± s. Sapma	Ort. (min. - maks.)	
Length of hospitalization	16,0 ± 13,6	13,0 (7,0–88,0)	16,9 ± 8,7	15,0 (5,0–49,0)	0,186
Operation time	214,7 ± 50,3	217,5 (110,0–300,0)	231,7 ± 33,3	240,0 (130,0–280,0)	0,156
Intraoperativehemorrhage	425,0 ± 155,8	400,0 (200,0–750,0)	497,8 ± 163,4	500,0 (250,0–900,0)	0,094
Survival time (months)	22,2 ± 14,5	19,5 (0,0–60,0)	18,1 ± 14,6	18,0 (0,0–51,0)	0,321

* Mann-Whitney U testwasusedforcomparision

There was no statistically significant difference observed in the median values of hospital length, operation time, intraoperative hemorrhage, and survival time between patients with sarcopenia and those without sarcopenia across various PMI cutoff values (*p* > 0.05) (Table 3).

**Table 3:** Comparison of hospitalization time, operation time, Intraop hemorrhage and survival time according to PMI cut-off value

	PMI cutoff				Pvalue*
	≥ 4,3		< 4,3		
	Mean ± Standard deviation	Mean(min. - maks.)	Mean ± Standard deviation	Mean(min. - maks.)	
Length of hospitalization	15,9 ± 12,9	13,5 (7,0–88,0)	17,2 ± 9,6	15,0 (5,0–49,0)	0,212
Operation time	218,2 ± 49,9	230,0 (110,0–300,0)	228,4 ± 32,0	220,0 (170,0–280,0)	0,418
Intraoperativehemorrhage	436,8 ± 164,3	400,0 (200,0–900,0)	489,5 ± 154,2	450,0 (250,0–750,0)	0,250
Survival time (months)	22,3 ± 15,1	19,0 (0,0–60,0)	17,1 ± 12,9	18,0 (0,0–44,0)	0,318

* Mann-Whitney U test was used for comparision

### Association with Clavien-Dindo classifications and Skeletal Muscle Index and Psoas Muscle Index

However, a statistically significant difference was found in the distribution of Clavien-Dindo classifications based on SMI cutoff values (*p* = 0.015). Specifically, 17.6% of patients with an SMI value of 38.5 and above and 47.8% of those with an SMI value below 38.5 were classified as Clavien-Dindo grade 3 or above. Regarding the distribution of survival statuses based on SMI cutoff values, no statistically significant difference was found (*p* = 0.127) (Table 4).

**Table 4:** Comparison of Clavien-Dindo and survival according to SMI cut-off value

	SMI Cut-off		Toplam	*P* value
	≥ 38,5	< 38,5		
Clavien-Dindo				
2 andbelow	28 (82,4)	12 (52,2)	40 (70,2)	**0**,**015**
3 andabove	6 (17,6)	11 (47,8)	17 (29,8)	
Survival				
Dead	20 (58,8)	18 (78,3)	38 (66,7)	0,127
Alive	14 (41,2)	5 (21,7)	19 (33,3)	

Similarly, a statistically significant difference was found in the distribution of Clavien-Dindo classifications based on PMI cutoff values (*p* = 0.008). Specifically, 18.4% of patients with a PMI value of 4.3 and above and 52.6% of those with a PMI value below 4.3 were classified as Clavien-Dindo grade 3 or above. However, no statistically significant difference was observed in the distribution of survival statuses based on PMI cutoff values (*p* = 0.164) (Table 5). There was no statistically significant difference in median survival times based on SMI (*p* = 0.093)and PMI (*p* = 0.063) (Table 6). A statistically significant weak positive correlation was observed between Body Mass Index (BMI) and SMI values (*p* = 0.024).

**Table 5:** Comparison of Clavien-Dindo and survival according to PMI cut-off value

	PMI cut-off		Total	*P* value
	≥ 4,3	< 4,3		
Clavien-Dindo				
2 ve aşağısı	31 (81,6)	9 (47,4)	40 (70,2)	**0**,**008**
3 ve üzeri	7 (18,4)	10 (52,6)	17 (29,8)	
Survival				
Dead	23 (60,5)	15 (78,9)	38 (66,7)	0,164
Alive	15 (39,5)	4 (21,1)	19 (33,3)	

**Table 6:** Comparison of survival time according to SMI and PMI cut-off values

	Mediansurvival (%95 CI)	Pvalue
SMI cut-off		
≥ 38,5	26 (18,052 − 33,948)	0,093*
< 38,5	21 (13,676 − 28,324)	
PMI cut-off		
≥ 4,3	26 (14,707 − 37,293)	0,063*
< 4,3	21 (13,667 − 28,333)	

*Logrank (Mantel-Cox) testi

### Association with Clavien-Dindo classifications and risk factors

Factors affecting survival were examined using univariate and multivariate Cox regression analysis. The analysis results indicated Clavien-Dindo classification as a risk factor in both univariate and multivariate models. After excluding four patients who died in the early postoperative period (Clavien-Dindo 5), the univariate model showed that the long-term mortality risk for patients with Clavien-Dindo 3 and above was 2.791 times higher compared to those with two or below. Similarly, in the multivariate model, after excluding Clavien-Dindo five patients, the mortality risk for patients with Clavien-Dindo 3 and above was 4.748 times higher compared to those with two or below. Other risk factors did not reach statistical significance (*p* > 0.05) (Table 7).

**Table 7:** Investigation of risk factors affecting survival time by Cox regression analysis

	Univariate		Multivariate	
	HR (%95 CI)	*P*	HR (%95 CI)	*p*
SMI Cutoff	1,741 (0,895–3,386)	0,102	1,193 (0,443–3,21)	0,727
PMI cutoff	1,866 (0,946–3,683)	0,072	1,38 (0,458–4,158)	0,567
FIGO	1,081 (0,768–1,522)	0,654	0,739 (0,407–1,341)	0,320
PeritonealCarcinomatosisindex	1,03 (0,951–1,114)	0,471	1,04 (0,904–1,196)	0,583
Residualtumor	1,085 (0,708–1,664)	0,708	1,04 (0,546–1,979)	0,906
Albumin	1,003 (0,997–1,008)	0,335	0,999 (0,994–1,004)	0,705
Chemotherapystatus	0,938 (0,55 − 1,601)	0,815	0,469 (0,213–1,03)	0,059
Clavien-DindoIndex	2,791 (1,377–5,657)	**0**,**004**	4,748 (1,645 − 13,707)	**0**,**004**

*Reference category

## Discussion

Sarcopenia, initially a condition that received relatively little attention when clinically defined, has become increasingly studied in recent years. Numerous studies have investigated its prognostic significance, particularly in pancreatic, hepatic, gastrointestinal, and urothelial cancers, but relatively fewer studies have focused on epithelial ovarian cancers [14–16].

In the study by Rutten et al. sarcopenic patients with advanced-stage ovarian cancer were reported to have significantly lower overall survival rates compared to non-sarcopenic patients. However, sarcopenia was not found to be decisive in overall survival and major complications in multivariate analysis [3]. Conversely, in the study by Kumar et al. no differences were observed in overall survival and disease-free survival between sarcopenic and non-sarcopenic groups of patients with advanced-stage ovarian cancer [18]. In our study, no significant difference in overall survival was observed between sarcopenic and non-sarcopenic groups. Similarly, Conrad et al. found no significant impact of sarcopenia on early postoperative morbidity in patients with advanced-stage ovarian cancer, but combined sarcopenia with hypoalbuminemia negatively affected overall survival [19]. However, in our study, no significant relationship was found between albumin levels and overall survival or postoperative early complications.

In contrast to these studies, Bronger et al. reported significantly lower overall survival and disease-free survival rates in sarcopenic patients with advanced-stage ovarian cancer [20]. Baseline sarcopenia was identified as a prognostic factor in advanced-stage ovarian cancer. Similarly, Huang et al. reported the effectiveness of sarcopenia in 5-year overall survival and disease-free survival rates in patients with advanced-stage ovarian cancer [21].

In the study by Ataseven et al. patients with advanced-stage ovarian cancer were classified based on quantitative SMI measurements using three different cut-off values. No association with overall survival was observed for any of the three cut-off values. However, qualitative assessment of sarcopenia based on muscle density measurement (Hounsfield Unit, HU) showed a significant association between low values and poor prognosis [22]. Silva de Paula et al. identified sarcopenic patients using quantitative SMI measurements and further categorized them qualitatively based on muscle density into High-Radiodensity and Low-Radiodensity SMI groups. Qualitative skeletal muscle status was reported as the most significant predictive factor for surgical complications. High-Radiodensity SMI was found to be an independent determinant of early surgical complications and associated with early mortality [8].

In the study by Kim et al. patients with advanced-stage ovarian cancer were grouped based on quantitative SMI measurements. No difference was found in overall survival and progression-free survival rates. Additionally, when patients were subgrouped based on Fat-to-Muscle Ratio (FMR), high FMR in sarcopenic patients was found to be unfavorable for overall survival, but no difference was observed in disease-free survival [23]. A meta-analysis conducted in 2020, which included most of these studies, found no significant relationship between the increase in treatment complications and sarcopenia based on data from five studies, but indicated an increased risk associated with sarcopenia. However, among the data from ten studies, a relationship was found between low overall survival and progression-free survival rates and sarcopenia, although the heterogeneity of data in these study groups was noted to be high [24].

In our study, patients were classified as sarcopenic and non-sarcopenic based on quantitative measurements. No differences were found in the characteristics of patients between the two groups. No statistically significant difference was observed in overall survival between sarcopenic and non-sarcopenic groups of patients who underwent PCS. However, major complications (Clavien-Dindo 3 and above) in the early postoperative period (first 30 days) were significantly more common in the sarcopenic group. Additionally, high PCI score and FIGO 3 C stage were associated with more early complications compared to low PCI score and FIGO 3 A-3B stage. The higher incidence of major complications in patients with high PCI score and FIGO 3 C stage is thought to be due to the higher rates of procedures such as diaphragmatic peritonectomy, diaphragm resection, splenectomy, extensive bowel resection, and liver resection. High PCI score and SMI < 38.5 were identified as risk factors for early postoperative complications in both univariate and multivariate analyses. However, no significant difference was found in overall survival.

Furthermore, when examining the factors affecting overall survival, the development of early major complications in the postoperative period was identified as a risk factor in both univariate and multivariate models. The mortality risk for patients with Clavien-Dindo 3 and above was found to be 2.791 times higher compared to those with 2 or below in the univariate model after excluding four patients who had died (Clavien-Dindo 5) in the early postoperative period. In the multivariate model, the mortality risk for patients with Clavien-Dindo 3 and above was 4.748 times higher compared to those with two or below. Delay or absence of adjuvant treatment in patients with major complications developing early postoperatively is considered to be the cause of this.

Our study has some limitations, such as being a retrospective review and having a small number of scanned patients. However, the patient group studied is quite homogeneous, and patients who underwent surgery at a single center were included in the study.

## Conclusion

Diagnosis of sarcopenia, measurement methods, and its effects on overall and disease-free survival in advanced-stage ovarian cancer are still areas of no consensus. However, determining patients’ sarcopenic status from routine preoperative CT images using quantitative measurements is a useful and cost-effective method. Determining a patient’s sarcopenic status before treatment is advantageous for predicting treatment complications. Providing immunonutrition and protein-rich nutritional support to sarcopenic patients in the preoperative period may contribute positively to the partial treatment of sarcopenia. However, since sarcopenia is a chronic process, it may take a long time to improve with nutritional support. In these patients who are at high risk for early postoperative complications, Interval Cytoreductive Surgery may be preferred over Primary Cytoreductive Surgery. During neoadjuvant chemotherapy, patients may be provided with nutritional support for a longer period of time, which may improve their sarcopenic status. Therefore, there is a need for prospective randomized studies with larger patient series.

## Data Availability

No datasets were generated or analysed during the current study.
